# Supplementary material for: Modeling glioblastoma heterogeneity as a dynamic network of cell states
Source: Mol Syst Biol. 2021 Sep 16;17(9):e10105. doi: 10.15252/msb.202010105 (PMC8444284; doi:10.15252/msb.202010105)
Supplement: Supplementary file 6 — Source Data for Figure 5 [file MSB-17-e10105-s004.zip › Figure5A_sourcedata/GSEA_3071/hallmarks_stateII.GseaPreranked.1623742536692/HALLMARK_REACTIVE_OXYGEN_SPECIES_PATHWAY.html]

Details for gene set HALLMARK\_REACTIVE\_OXYGEN\_SPECIES\_PATHWAY[GSEA]

|  || Dataset | state23071 |
| Phenotype | NoPhenotypeAvailable |
| Upregulated in class | na\_neg |
| GeneSet | HALLMARK\_REACTIVE\_OXYGEN\_SPECIES\_PATHWAY |
| Enrichment Score (ES) | -0.17589134 |
| Normalized Enrichment Score (NES) | -0.6719185 |
| Nominal p-value | 0.86907023 |
| FDR q-value | 0.94042563 |
| FWER p-Value | 1.0 |
Table: GSEA Results Summary

  

Fig 1: Enrichment plot: HALLMARK\_REACTIVE\_OXYGEN\_SPECIES\_PATHWAY      
 Profile of the Running ES Score & Positions of GeneSet Members on the Rank Ordered List

  

| PROBE | GENE SYMBOL | GENE\_TITLE | RANK IN GENE LIST | RANK METRIC SCORE | RUNNING ES | CORE ENRICHMENT || 1 | PFKP |  |  | 136 | 0.988 | 0.0568 | Yes |
| 2 | MGST1 |  |  | 235 | 0.838 | 0.1093 | Yes |
| 3 | PDLIM1 |  |  | 601 | 0.578 | 0.0726 | Yes |
| 4 | NQO1 |  |  | 732 | 0.522 | 0.0884 | Yes |
| 5 | GLRX2 |  |  | 907 | 0.469 | 0.0886 | Yes |
| 6 | CDKN2D |  |  | 911 | 0.468 | 0.1306 | Yes |
| 7 | TXNRD2 |  |  | 974 | 0.450 | 0.1565 | Yes |
| 8 | TXN |  |  | 1087 | 0.419 | 0.1673 | Yes |
| 9 | TXNRD1 |  |  | 1269 | 0.383 | 0.1579 | Yes |
| 10 | G6PD |  |  | 1346 | 0.368 | 0.1729 | Yes |
| 11 | PRDX4 |  |  | 1470 | 0.344 | 0.1742 | Yes |
| 12 | GCLM |  |  | 1889 | 0.287 | 0.0979 | No |
| 13 | CAT |  |  | 2344 | -0.264 | 0.0107 | No |
| 14 | LAMTOR5 |  |  | 2821 | -0.339 | -0.0751 | No |
| 15 | GLRX |  |  | 2834 | -0.342 | -0.0468 | No |
| 16 | MSRA |  |  | 2891 | -0.354 | -0.0283 | No |
| 17 | PRNP |  |  | 3383 | -0.491 | -0.1038 | No |
| 18 | NDUFB4 |  |  | 3678 | -0.668 | -0.1149 | No |
| 19 | GPX4 |  |  | 3915 | -0.986 | -0.0828 | No |
| 20 | PRDX2 |  |  | 4021 | -1.396 | 0.0189 | No |
Table: GSEA details [plain text format]

  

Fig 2: HALLMARK\_REACTIVE\_OXYGEN\_SPECIES\_PATHWAY: Random ES distribution      
 Gene set null distribution of ES for **HALLMARK\_REACTIVE\_OXYGEN\_SPECIES\_PATHWAY**
